# On the accuracy of short-term COVID-19 fatality forecasts

**DOI:** 10.1186/s12879-022-07205-9

**Published:** 2022-03-14

**Authors:** Nino Antulov-Fantulin, Lucas Böttcher

**Affiliations:** 1 Computational Social Science, ETH Zurich, 8092 Zurich, Switzerland; 2 Computational Social Science, Frankfurt School of Finance and Management, 60322 Frankfurt, Germany; 3 Department of Computational Medicine, University of California, Los Angeles, Los Angeles, 90095-1766 USA

**Keywords:** COVID-19, Forecasting, Numerical analysis, Computer-assisted, Epidemiological monitoring

## Abstract

**Background:**

Forecasting new cases, hospitalizations, and disease-induced deaths is an important part of infectious disease surveillance and helps guide health officials in implementing effective countermeasures. For disease surveillance in the US, the Centers for Disease Control and Prevention (CDC) combine more than 65 individual forecasts of these numbers in an ensemble forecast at national and state levels. A similar initiative has been launched by the European CDC (ECDC) in the second half of 2021.

**Methods:**

We collected data on CDC and ECDC ensemble forecasts of COVID-19 fatalities, and we compare them with easily interpretable “Euler” forecasts serving as a model-free benchmark that is only based on the local rate of change of the incidence curve. The term “Euler method” is motivated by the eponymous numerical integration scheme that calculates the value of a function at a future time step based on the current rate of change.

**Results:**

Our results show that simple and easily interpretable “Euler” forecasts can compete favorably with both CDC and ECDC ensemble forecasts on short-term forecasting horizons of 1 week. However, ensemble forecasts better perform on longer forecasting horizons.

**Conclusions:**

Using the current rate of change in incidences as estimates of future incidence changes is useful for epidemic forecasting on short time horizons. An advantage of the proposed method over other forecasting approaches is that it can be implemented with a very limited amount of work and without relying on additional data (*e.g.*, data on human mobility and contact patterns) and high-performance computing systems.

## Background

Over the course of the COVID-19 pandemic more than 65 international research groups contributed to an ensemble forecast of reported COVID-19 cases, hospitalizations, and fatalities in the US [1]. These forecasts are a central source of information on the further development of the pandemic and used by various governmental and non-governmental entities including the Centers for Disease Control and Prevention (CDC) [2]. A similar initiative has been launched in by the European CDC (ECDC) in the second half of 2021 [3].

Different forecasting methods [4, 5] rely on different underlying models and assumptions. One may roughly divide forecasting models into three different classes: (i) mechanistic models [6, 7], (ii) purely data-driven models [8], and (iii) hybrid models. Most classical epidemic models are mechanistic and aim at describing disease dynamics in terms of interacting individuals in a population. Such models are usually applied to describe the influence of certain factors (e.g., population density, demographics, contact patterns, mobility, etc.) on the dynamics of an epidemic. Data-driven and machine learning models make fewer assumptions about the underlying dynamics and are applicable to a broader range of forecasting problems, but they also come at the cost of less interpretability for policymakers and epidemiologists.

Here, we show that a very basic, model-free forecasting approach provides effective short-term forecasts of COVID-19 fatalities. We refer to this method as “Euler forecast” because of its mathematical connection to the Euler method [9, 10] that is used in computational mathematics to calculate the value of a function at a future time step based on the current rate of change.

**Fig. 1:**
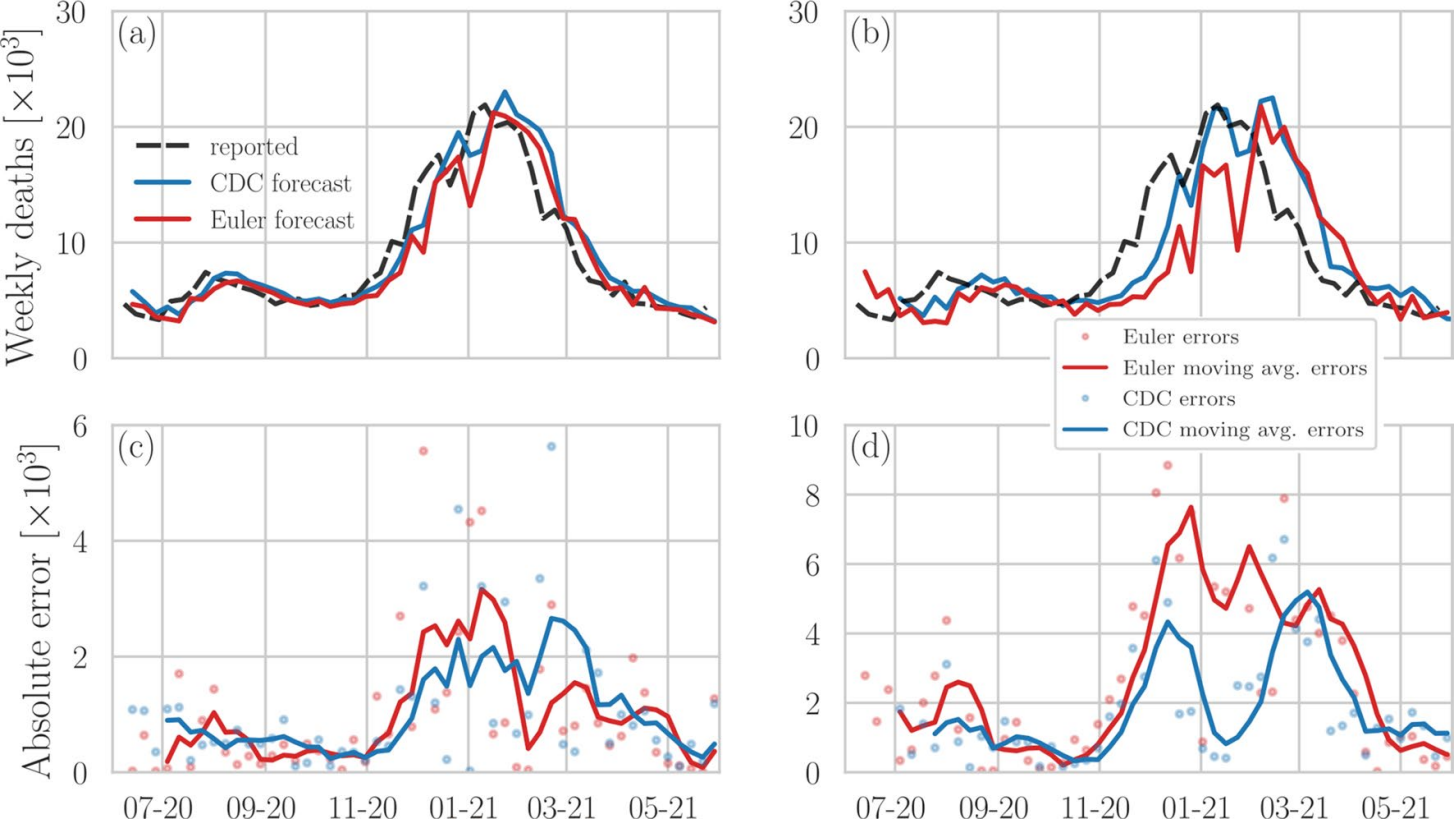
Comparison of predicted and reported weekly COVID-19 deaths in the US (data updated in June 2021). **a**, **b** Forecasts of reported weekly COVID-19 deaths in the US for **a** 1-week and **b** 4-week forecasting horizons. Blue and red lines represent CDC ensemble forecasts [1] and regularized Euler forecasts [Eq. (3)] with $\uplambda =10$, respectively. Reported COVID-19 fatalities (dashed black lines) are based on [11]. **c**, **d** 4-week moving averages of weekly forecasting errors of Euler–Lagrange and CDC ensemble forecasts. Solid lines indicate 4-week moving averages that are calculated based on the shown data points

## Methods

Different epidemiological models [12] capture different aspects of disease spread and many of these models are based on coupled ordinary differential equations (ODEs). In the susceptible-infected-recovered (SIR) model [6], the rate of change of *S*(*t*), the number of susceptible individuals at time *t*, is described by the ODE

1 $$\begin{aligned} {\dot{S}}(t)= -\beta S(t)I(t)/N. \end{aligned}$$

Here, *I*(*t*) and *N* denote the number of infectious individuals at time *t* and the population size, respectively. The infection rate is $\beta$.

We now assume that the epidemic state of a population can be represented by some quantity *y*(*t*) and that its evolution (*i.e.*, the rate of change) is described by a function *g*(*y*(*t*), *t*). That is,

2 $$\begin{aligned} {\dot{y}}(t)= g(y(t),t). \end{aligned}$$

The SIR model (1) can be written in terms of Eq. (2) by setting $y(t)=(S(t), I(t), R(t))^\top$.

Euler’s method [9] is one of the simplest numerical procedures for solving ordinary differential equations of the form (2) for a given initial condition. This method uses a timestep $\Delta t>0$ to approximate the solution of Eq. (2) at times $t_1, t_2, \dots , t_n$ according to^9^, ^10^

3 $$\begin{aligned} y(t_{n+1})= y(t_{n}) + \Delta t {\dot{y}}(t_n). \end{aligned}$$

However, in reality the functional form $g(\cdot )$ that describes the rate of change of *y*(*t*) that is relevant for infectious disease surveillance is usually not known. In the following paragraphs, we thus describe practical ways how to estimate COVID-19 fatalities *y*(*t*) and their local rate of change ${\dot{y}}(t)$ from noisy observation data.

We first collected data on CDC ensemble forecasts between June 2020 and June 2021 [1].^1^ Ensemble forecasts are available for cumulative and weekly incidence and fatality numbers and a forecasting horizon between 1 to 4 weeks. All forecasts use data from the Johns Hopkins Coronavirus Resource Center [11] as ground truth. Forecasts are made for epidemiological weeks which run Sunday through Saturday. As an example, if forecasts with 1- and 4-week forecasting horizons are being made on June 8, 2020 the corresponding forecasting intervals are June 7–June 13, 2020 and June 7–July 4, 2020 [13].

We compare CDC and ECDC ensemble forecasts of COVID-19 fatalities with a simple and easily interpretable forecasting method. To do so, let *y*(*t*) be the incidence of COVID-19 fatalities at time *t*. We use ${\dot{y}}(t)$ to denote the rate of change of *y*(*t*) at time *t*. Forecasting the incidence $y(t+\Delta t)$ at a target time $t+\Delta t$ requires us to find an estimate of this quantity at an earlier time *t*. A straightforward way to construct short-term forecasts is to (i) use the current rate of change ${\dot{y}}(t)$ and (ii) determine a forecast at time $t_k=t_0+k\Delta t$ according to the Euler method [9, 10]

4 $$\begin{aligned} y(t_0+k\Delta t)= \underbrace{y(t_0)}_{\text {last incidence}} + \underbrace{k\Delta t\,{\dot{y}}(t_0)}_{\text {incidence correction}}, \end{aligned}$$

where $\Delta t$ and $k=1,2,\dots$ represent a time step (e.g., 1 week) and the number of time steps in the forecasting horizon, respectively. However, observed incidences are subject to observation noise that results from confounding factors including sampling bias, measurement errors, and reporting delays [14].

**Fig. 2:**
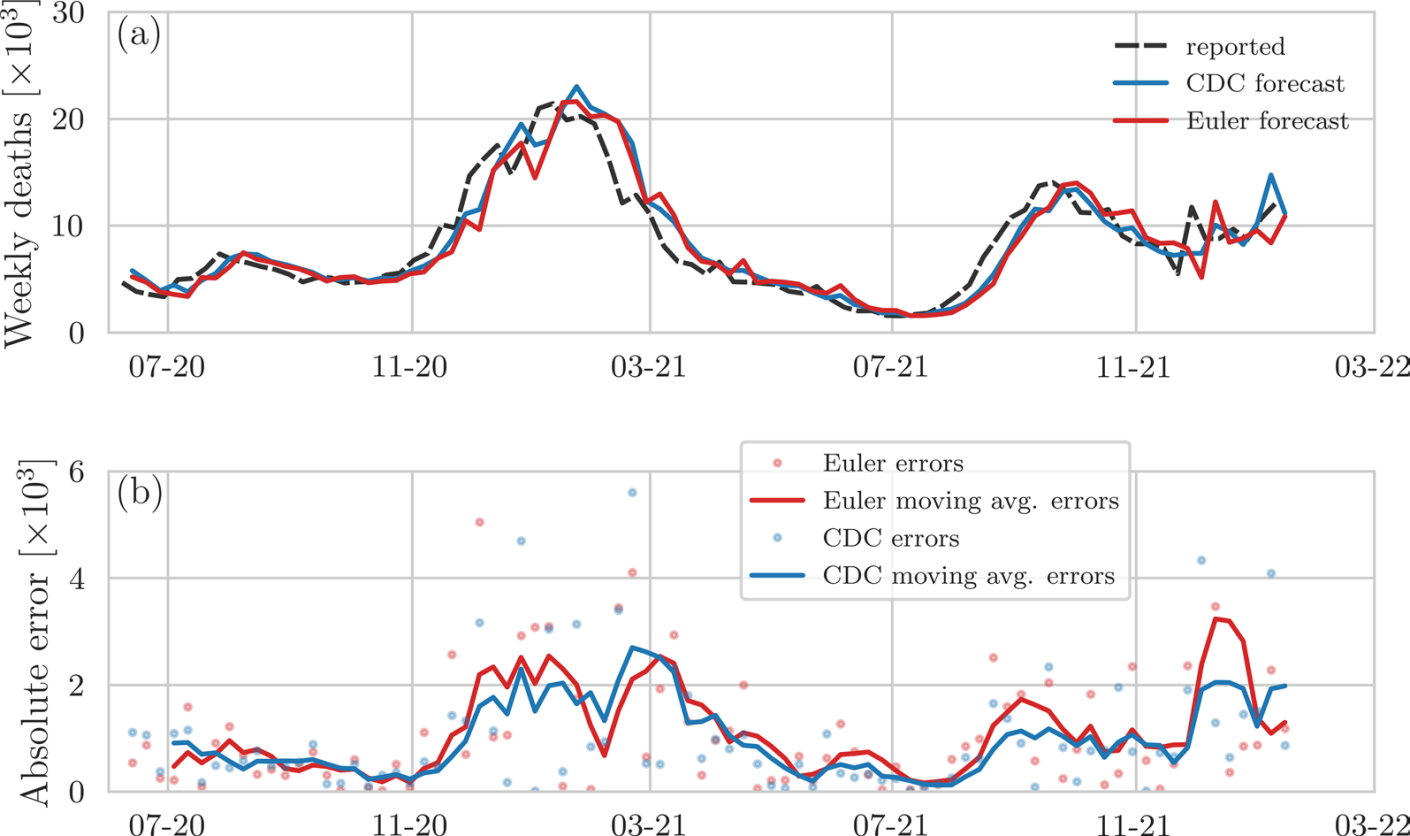
Comparison of predicted and reported weekly COVID-19 deaths in the US (data updated in January 2022). **a** Forecasts of reported weekly COVID-19 deaths in the US for 1-week forecasting horizons. Blue and red lines represent CDC ensemble forecasts [1] and regularized Euler forecasts [Eq. (6)] with $\uplambda =10^5$, respectively. Reported COVID-19 fatalities (dashed black lines) are based on [11]. **b** 1-week moving averages of weekly forecasting errors of Euler–Lagrange and CDC ensemble forecasts. Solid lines indicate 1-week moving averages that are calculated based on the shown data points

**Fig. 3:**
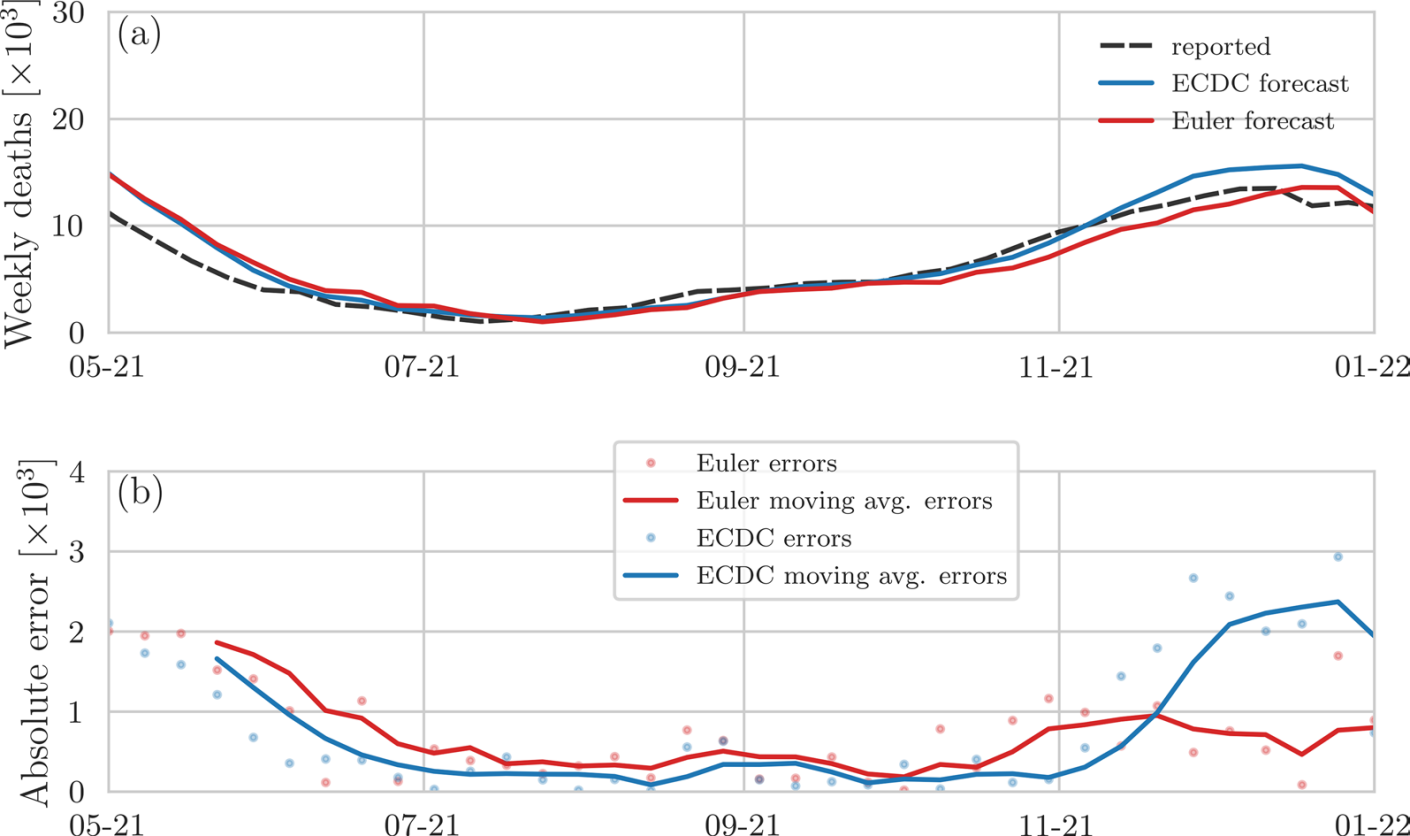
Comparison of predicted and reported weekly COVID-19 deaths in EU and EFTA countries and the UK (data updated January 2022). **a** Forecasts of reported weekly COVID-19 deaths in EU and EFTA countries and the UK for 1-week forecasting horizons. Blue and red lines represent ECDC ensemble forecasts [3] and regularized Euler forecasts [Eq. (6)] with $\uplambda =10^4$, respectively. Reported COVID-19 fatalities (dashed black lines) are based on [11]. **b** 1-week moving averages of weekly forecasting errors of Euler–Lagrange and ECDC ensemble forecasts. Solid lines indicate 1-week moving averages that are calculated based on the shown data points

A possible way to “de-noise” observed data is to use weekly incidences instead of daily incidence levels. If observational noise can be reduced by averaging over a period of several days, daily errors are less pronounced on a weekly level. However, the local daily derivative is quite sensitive to noise and our incidence correction term may not help in making accurate short-term forecasts. Therefore, we can impose some degree of regularity to reduce the level of noise with the following minimization

5 $$\begin{aligned} {{\,\mathrm{arg\,min}\,}}_{\{w_k\}} \sum _k (y_k-w_k)^2 + \uplambda \sum _k (w_k-w_{k-1})^2, \end{aligned}$$

where $y_k=y(t_0+k\Delta t)$, $w_k=w(t_0+k \Delta t)$ is a regularized approximation of $y_k$, and $\uplambda$ is a regularization parameter. In the limit $\uplambda \rightarrow 0$, the argument of Eq. (5) is minimized if *w*(*t*) approaches *y*(*t*). In the limit $\uplambda \rightarrow \infty$, the argument of Eq. (5) is minimized if *w*(*t*) is constant (i.e., if $w_k-w_{k-1}=0$). This optimization process has its equivalent Euler–Lagrange formulation for numerical differentiation [15, 16]. Values of $\uplambda \in (0,\infty )$ yield functions *w*(*t*) that are smoothed versions of *y*(*t*) with respect to the discrete rate of change $w_k-w_{k-1}$. Finally, the regularized Euler short-term forecast^2^ is given by

6 $$\begin{aligned} y(t+k\Delta t)= y(t) + k\,[w(t)-w(t-\Delta t)]. \end{aligned}$$

In the following section, we use both the standard Euler method and the regularized Euler method to generate forecasts of reported COVID-19 fatalities.

Our source codes are publicly available at [17].

## Results

Figure 1 shows CDC ensemble forecasts (solid blue lines) of the weekly incidences of reported COVID-19 fatalities from June 2020 until June 2021. The dashed black lines indicate reported COVID-19 fatalities. Between June and early November 2020, the majority of reported fatalities were close to the ensemble forecast. As COVID-19 deaths surged in November 2020, the forecasts of the ensemble method became less accurate than in previous months.

For a comparison between the CDC ensemble point estimates and those obtained with the regularized Euler method [Eq. (6)], Fig. 1 also shows regularized Euler forecasts (solid red lines) of weekly incidences of COVID-19 fatalities in the US. We observe that 1-week CDC ensemble forecast for the majority of data points are not more accurate than 1-week Euler forecasts (Fig. 1a), which we use as a local-derivative-based forecasting benchmark. Although Euler and CDC forecasts still exhibit a similar structure for a 4-week forecasting horizon (Fig. 1b), the Euler method is associated with larger deviations from the reported fatalities than the CDC ensemble method. To quantify differences in forecasting errors between the two methods, we use

7 $$\begin{aligned} \delta _{x,y}(t)=|x(t)-y(t)| \end{aligned}$$

to denote the absolute error between the Euler or CDC forecast *y*(*t*) and the ground truth *x(t)* at time *t*.

Figure 1c, d show the 4-week moving averages of weekly forecasting errors $\delta (t)$ (solid lines) of the Euler method (red) and the CDC ensemble (blue) method. As suggested by our above discussion of Fig. 1a, we observe that the error of the Euler method is substantially smaller than that of the ensemble forecast for a 1-week forecasting horizon. In about 61% of the forecasting instances shown in Fig. 1a, the regularized Euler method has a smaller error than the CDC ensemble forecast. The cumulative forecasting errors are 49,925 (Euler) and 52,885 (CDC). Without correction term $k\,[w(t)-w(t-\Delta t)]$ in Eq. (6), the cumulative forecasting error of the Euler method is 52,660, again smaller than that of CDC ensemble forecast. Note that forecasts without correction correspond to a simple shift of the incidence curve [see Eq. (4)]. For a 4-week forecasting horizon (Fig. 1d), the cumulative error of the CDC ensemble forecast is 87,717, about 35% smaller than that of the Euler method.

To complement our analysis of CDC ensemble forecasts from June 2020 until June 2021, we have updated the CDC ensemble forecast data in January 2022. We conduct a separate analysis because historical ensemble forecasts can be changed a posteriori [18]. In addition, we have also gathered ECDC forecasts from May 2021 until January 2022 [3] for EU and EFTA countries and the UK.

Based on the second set of CDC ensemble forecasts, we observe that the accuracy of 1-week ensemble forecast (Fig. 2a, b) improved slightly with respect to the regularized Euler forecast. The cumulative for ecasting errors until January 2022 are 93,645 (Euler regularized) and 84,870 (CDC). Without correction term $k\,[w(t)-w(t-\Delta t)]$ in Eq. (6), the cumulative forecasting error of the Euler method is 94,108. For a 1-week forecasting horizon, the Euler method is associated with larger deviations from the reported fatalities than the CDC ensemble method i n about 51% of the forecasting instances shown in Fig. 2. On a longer forecasting horizo n of 4 weeks, the Euler method performs better than the CDC ensemble method in only 16% of all cases. This result is not surprising because the Euler method relies on smoothed curve shift and is not designed for longer forecasting horizons. For the comparison with ECDC [3] ensemble forecasts, we use all data that was available in January 2022 to compare the forecasting errors with those of Euler forecasts (Fig. 3a, b). The cumulative forecasting errors until January 2022 are 28,769 (Euler regularized) and 30,942 (ECDC). Without correction term $k\,[w(t)-w(t-\Delta t)]$ in Eq. (6), the cumulative forecasting error of the Euler method is 30,353. In about 36% of the forecasting instances shown in Fig. 3a, the regularized Euler method has a s maller error than the ECDC ensemble forecast. Finally, in Appendix Figs. 4 and 5 we show joint comparisons of errors of Euler, regularized Euler, and (E)CDC forecasts.

## Discussion

On 1-week forecasting horizons, regularized Euler forecasts have smaller errors with respect to CDC ensemble forecasts in about 61% of all cases up to June 2021 and in about 49% of all cases up to January 2022. The cumulative errors are worse for CDC up to June 2021 and better if we consider data up to January 2022. In comparison with ECDC forecasts, the regularized Euler method performs better in 36% of the forecasting instances on a 1-week forecasting horizon, while ECDC forecasts are associated with a lower cumulative error up to January 2022. Overall, on a 1-week forecasting horizon simple Euler forecasts can perform similarly to ensemble methods that are composed of a large number of more complex models. In agreement with [19], our results emphasize the importance of benchmarking complex forecasting models against simple forecasting baselines to further improve forecasting accuracy. Similar conclusions were drawn in a recent study [19] that compared Euler-like forecasts with those generated by Google Flu Trends. Our study also points towards recent findings on algorithm rejection and aversion [20] that found that “people have diminishing sensitivity to forecasting error” and that “people are less likely to use the best possible algorithm in decision domains that are more unpredictable”. Finally, in highly uncertain and noisy forecasting regimes, simple methods tend to outperform more complex methods because of a more favorable bias-variance trade-off [21].

## Conclusions

Our results suggest that easily interpretable methods like the Euler method, a model-free local-derivative-based forecasting benchmark, provide an effective alternative to more complex epidemic forecasting frameworks on short-term forecasting horizons. Simple curve shifts without regularization provide forecasts that are close to CDC and ECDC ensemble forecast, a finding that can help improve existing forecasting methods. For longer forecasting horizons, it is not surprising that CDC and ECDC forecasts that rely on additional input data and epidemiological and statistical models become more accurate than Euler-like forecasting benchmarks. One clear advantage of Euler forecasting methods is that they are less labor and resource intensive than more complex forecasting models, which often rely on the knowledge of expert groups and require specialized computing infrastructure. In their simplest implementation, Euler forecasts use the currently observed incidence rate as an estimate of the incidence rate in the following week. Regularization methods (6) can help further improve such data-driven forecasts.

## Figures and Tables

**Fig. 4:**
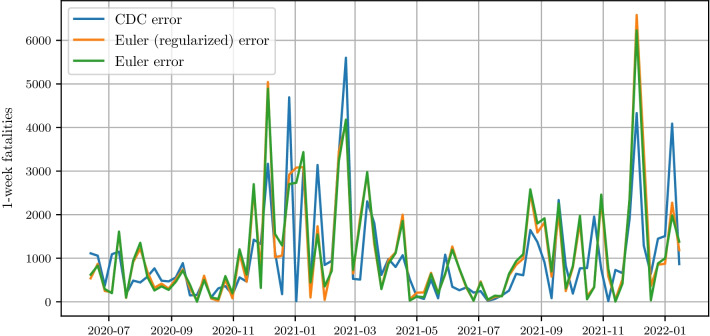
Forecasting errors of Euler, regularized Euler, and CDC ensemble forecasts (data updated in January 2022)

**Fig. 5:**
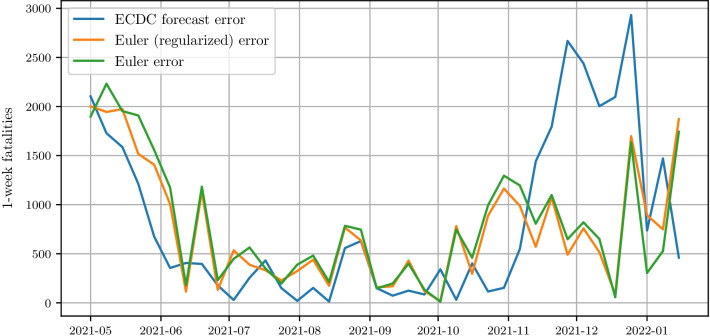
Forecasting errors of Euler, regularized Euler, and ECDC ensemble forecasts (data updated in January 2022)

## Data Availability

All data used in this article are available at [1, 11]. Our source codes are publicly available at [17].

## Appendix

See Figs. 4 and 5.

## Abbreviations

CDC: The Centers for Disease Control and Prevention
COVID-19: Coronavirus disease of 2019

## Footnotes

1 Additional results for CDC and ECDC forecasts that are based on data that was last updated in January 2022 are provided in the Results section.

2 All optimization procedures in this work were robust to regularization parameter selection and were applied in a causal manner. That is, at the prediction time *T* only historical data $y(t\le T)$ is being used in the minimization (5).
